# Evaluation of *About Being Active*, an online lesson about physical activity shows that perception of being physically active is higher in eating competent low-income women

**DOI:** 10.1186/1472-6874-13-12

**Published:** 2013-03-13

**Authors:** Barbara Lohse, Kristen Arnold, Patricia Wamboldt

**Affiliations:** 1 Department of Nutritional Sciences, The Pennsylvania State University, 205 Chandlee Lab, University Park, PA, 16802, USA; 2 Department of Nutritional Sciences, The Pennsylvania State University, 306 Chandlee Lab, University Park, PA, 16802, USA

**Keywords:** Eating behavior, Eating competence, Education, Low-income, Physical activity, Women

## Abstract

**Background:**

Eating competence (EC) has been associated with positive health outcomes such as reduced cardiovascular risk and higher diet quality. This study compared reported physical activity and EC in 512 low-income women participating in an online program that included a physical activity lesson and assessed response to this lesson.

**Methods:**

Educational intervention and surveys were completed online. EC was assessed with the Satter Eating Competence Inventory for Low-Income (ecSI/LI).

**Results:**

Participants were mostly white, <31 years, overweight/obese (60%), and food insecure (58%). EC was higher for those who self-reported being physically active (30.1 ± 8.3 vs. 24.9 ± 8.1; P<0.001) and were active for ≥ 30 minutes/day (29.9 ± 8.3 vs. 26.3 ± 8.6), even with age, weight satisfaction, and BMI controlled. EC of obese physically active persons was higher than normal weight, but physically inactive women. The physical activity module was well received with responses unrelated to time involved or physical activity level.

**Conclusions:**

Low-income women were interested in learning about physical activity and responded positively to online delivery. Overall EC levels were low, but higher for physically active women, supporting efforts to enhance EC. Additional research is needed to determine if EC is associated with responses to physical activity education.

## Background

Eating competence (EC) has been described by the Satter Eating Competence Model (ecSatter) as an intra-individual approach to eating and food-related attitudes and behaviors that entrains positive bio-psychosocial outcomes [1]. The gestalt of ecSatter eschews traditional detail on portion sizes, specific foods or nutrients, but rather advocates for nutrition education that emphasizes eating enjoyment; internal regulation of intake and letting body weight be dictated by lifestyle and genetics; using skills to provide meals regularly; and eating a variety of foods for pleasure, rather than just to meet dietary guidelines [2]. Therefore, it is of interest that studies have shown that competent eaters have a higher diet quality [3,4]; fewer risks for cardiovascular disease including lower blood pressure, lower LDL-cholesterol and increased HDL-cholesterol [4,5]; lower BMI, greater weight satisfaction; better developed food resource management skills [6-8]; higher sleep quality [9] and fewer correlates of disordered eating, e.g., emotional eating, drive for thinness, interpersonal insecurity, and maturity fears [6,7]. These relationships have been noted in samples varied by gender, age, and socioeconomic status.

Competent eaters are more likely to be physically active. In a multi-state study of university students (n=997) EC predicted being moderately and vigorously active as assessed by the International Physical Activity Questionnaire (IPAQ) and EC was significantly associated with VO2max (Unpublished observations; Greene GW, White AA, Hoerr SL, Lohse B, Schembre SM, Riebe D, Patterson J, Kattelmann KK, Shoff S, Horacek T, Blissmer B, Phillips BW). In addition to this association with objective measures, competent eaters are more likely to self-report being physically active [6-8].

Being physically active was associated with several positive health benefits in a sample of 506 low-income women. Those perceiving themselves to be physically active (which was 51% of the sample), were more likely to be of normal weight (P < 0.001) and satisfied with their weight (P<0.001) [7]. The finding that low-income women who are eating competent are significantly more likely than those not eating competent to report being physically active prompts further study of this phenomenon because low-income audiences, specifically women, have been shown to be disproportionally more likely to be inactive [10].

For example, approximately 32% of the population below 100% of the poverty level met the guidelines from the federal 2008 Physical Activity Guidelines for Americans, compared to 53% of the population at or above 200% of the poverty level [11,12]. Low physical activity levels among low-income women are disconcerting because regular physical activity reduces the risk of cardiovascular disease, type 2 diabetes, and obesity [10]. Yet, low-income women report numerous barriers, such as fatigue, culture, health problems, absence of child care, and lack of encouragement, that make it difficult for them to meet the physical activity recommendations [13-15]. Focus groups have revealed that low-income women have many misconceptions regarding physical activity that prevent them from meeting the recommendations. Hoebeke [14] suggests that these misconceptions could be tempered by promoting education about physical activity. For example, educating women who experience fatigue that physical activity may lessen the influence of fatigue as a barrier to meeting the physical activity recommendations. Low-income women experience barriers that differ from those of the general population, such as limited time and resources, stressing the need for tailored interventions. Interventions that aim to educate Americans on the importance of physical activity have been developed however, few target low-income women [14]. A program utilizing community health workers to deliver WISEWOMAN adapted for low-income Latinas demonstrated that low-income women with limited education respond to culturally tailored education by increasing physical activity or readiness to engage in physical activity [16].

Web-based education on physical activity has not been tested as a means of increasing physical activity in low-income adults. However, web-based education is feasible for low-income populations and internet access is widespread among low-income persons. For example, a study of 1,620 participants in the Indiana Family Nutrition Program, which targets persons eligible for participation on the Supplemental Nutrition Assistance Program (SNAP) Education (SNAP-Ed), revealed that 50% owned a computer, 78% of those owning a home computer accessed the internet from home, and 34% used the internet for nutrition information searches [17]. This suggests an increase in internet access of low-income Americans because an earlier report by the Pew Internet and American Life project reported that 65% of low-income adults (annual income < $30,000) had internet access [18]. Another study, utilizing face-to-face interviews with low-income adults in Pennsylvania indicated that 80% of study participants had access to the internet, and used the internet as their main resource for assessing nutrition and other health information [19]. A high level of internet access was also noted in a sample of rural, low-income adult women in Maryland, with more than 80% reporting use of the internet to access health and educational information [20].

Therefore, the purposes of this study were to 1) describe EC and socio-demographic characteristics of low-income women and compare these between women who perceive being physically active with those who don’t and 2) to examine their responses to an online physical activity lesson designed to foster awareness of and attention to a physically active lifestyle for low-income audiences.

## Methods

### Research design

**Figure 1:**
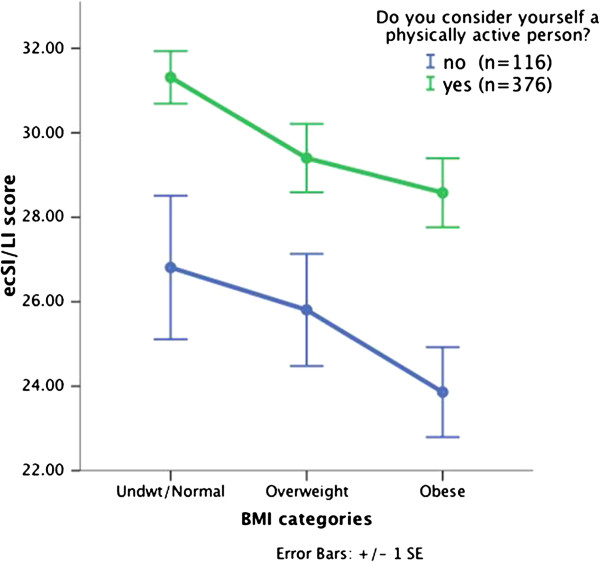
Study design, recruitment, and participation patterns.

This descriptive study utilized baseline and lesson evaluation data gathered from a randomized, controlled impact assessment of *About Eating*[21]. As shown in Figure 1, this online nutrition education program consisted of 4 lessons addressing the dynamics of EC (eating attitudes and behaviors, food acceptance, internal regulation, and eating contexts) and 1 lesson (entitled *About Being Active*) focused on physical activity.

### Development and delivery of *About Being Active*

Key features of *About Being Active* were present in 2 online lessons that were included in a 10-lesson course that was developed for and tested with undergraduate college students [22]. Several lessons in this course were translated to a low-income audience and pilot-tested [23,24]. The physical activity lessons were also adapted for a low-income audience in 2 stages. First, utilizing a “talk-aloud” process, cognitive interviews were conducted with 12 low-income women (mean age 25.3 years; range 19 – 38 years) in WIC clinics located in 3 non-contiguous Pennsylvania counties to assess their acceptance and understanding of lesson content and design. Interviews lasted an average of 37.8 minutes and ranged from 20 to 71 minutes in length. Participants provided feedback about graphics, readability, comprehension, and interest in the physical activity lessons. In addition, they verbalized perceived barriers to activity. As a result of interview findings, concepts from 2 lessons were merged into 1 lesson and more barriers to physical activity (e.g. sick children, lack of time, fatigue) were addressed. Interactive components were retained. The revised lesson was made available online to 9 respondents from the first stage who expressed interest in providing comments after revision. The emergent lesson (which can be viewed at www.needscenter.org/about-eating), provided strategies for increasing physical activity, managing obstacles to physical education, and setting activity goals. A detailed description of lesson components is shown in Table 1. The web-based lesson aimed to meet participants at their current level of activity, and encouraged increasing activity time and intensity to maximize health benefits.

**Table 1:** ***About being active *description**

**Lesson components**	**Specific content examples**
**Exercise IQ survey**	Participants answer “true” or “false” to these statements:
	• Doing a lot of sit-ups will give me a flat stomach
	• When it comes to exercise, the saying ‘no pain, no gain’ is…
	• While exercising, feeling thirsty is a sign that you need to drink fluids (such as water)
	• Body weight can stay the same or increase with exercise even though you are losing fat
	• If you can say a few words, catch your breath and then carry on talking while exercising, you are exercising at a good level for you
	• You need to use sports drinks during any exercise
**Typical patterns of physical activity and inactivity**	Think about your typical day. Which person are you most like?
	• Sit Down Sarah- Sarah isn’t active during the day. She sleeps, eats, takes the bus or drives to work and to do errands, mostly sits at her desk or computer station at work, watches TV, mostly plays video games for fun and then goes to bed.
	• Hardcore Hayley- Hardcore Hayley isn’t much more active during the day than Sit-down Sarah. Like Sarah, Hayley sits a lot during the day, takes the bus or drives everywhere, but she does take time to work out for about an hour each day. Hayley isn’t concerned about her sit-down lifestyle because she works out each day.
	• Lifestyle Linda- Lifestyle Linda is active throughout the day. Linda may walk or ride a bike to work or when doing errands. If she drives, she parks the car at the far end of the parking lot so she can walk to where she is going. Linda doesn’t necessarily work out or exercise regularly but she gets a lot of activity during the day. She ends up burning as many calories as Hardcore Hayley whose only activity is a regular exercise session.
	• Combo Chris- Combo Chris is not only active during the day, but she also finds time for a serious exercise session. Chris has the highest calorie use of all and gets the most benefit from being active.
**What are your reasons for being more active?**	For each response, a pop-up window appears to describe how exercise will lead to each of these goals
**Dealing with obstacles**	When you select an obstacle, a pop-up describes ways to overcome these obstacles and find ways to exercise despite the obstacles.
**Setting goals**	• Asks participants to write down exercise goals
	• Provides a grid to record activity throughout the day
**Feel good about moving**	Stresses the importance of feeling good about moving and resting your body as fitness goals.

For most participants, order of lesson completion was learner-driven. However, as part of a sub-study to assess the four lessons that focused on each EC precept, half of the persons who were active < 30 minutes a day were randomly assigned to a group who was required to complete the post-intervention assessment before completing *About Being Active*, thus requiring this lesson to be available last.

### Recruitment and data collection

Participants were recruited from Pennsylvania counties not participating in SNAP-Ed programs using two strategies, both of which directed interested persons to an identical website. One strategy included posting flyers in low-income venues such as job training centers, laundromats, housing assistance offices, and libraries. A second recruitment plan utilized addresses and phone numbers of SNAP participants supplied by the Pennsylvania Department of Public Welfare to make recruitment calls and to send informative postcards. As shown in Figure 1, website responders completed an eligibility survey prior to study inclusion to reach women between the ages of 18-45 who were English literate, had internet access, and lived in one of the 40 Pennsylvania counties with no or very limited (i.e., only indirect education in the County Assistance Office waiting area) participation in Supplemental Nutrition Assistance Program (SNAP) Education (SNAP-Ed). Persons with poor health (e.g. diagnosis of cancer or heart disease within the past 5 years), who studied nutrition full-time or were employed as a nutritionist were excluded. Online surveys (Qualtrics; Provo, UT) administered at the start of the study and after completion of *About Being* provided qualitative and quantitative responses.

Baseline survey items included demographics; tested queries about health, weight satisfaction (1= very satisfied, 5=very unsatisfied), desired weight loss, and worry about money for food (1=always, 5=never); the Satter Eating Competence Inventory for Low-Income (ecSI/LI); and the Adult Food Security Screener of the United States Department of Agriculture (USDA).

ecSI/LI is a 16-item likert scaled inventory with possible scores of 0 – 48; higher scores indicate greater EC. Construct validity and internal reliability has been established in a sample of low-income, pre-menopausal women in Pennsylvania [7]. The ecSI/LI consists of 4 subscales: Eating attitudes and behavior (5 items, possible score of 0 – 15); internal regulation (3 items, possible score 0 -9); Food acceptance (3 items, possible score 0 – 9), and contextual skills (5 items, possible score of 0 – 15.)

The USDA Adult Food Security Screener [25] is a tested survey that identifies food security at the household level. Affirmative responses to the 10-items are summed to a raw score, which is further categorized as high, marginal, low, or very low food security. Respondents with scores denoted as high or marginal are classified as food secure; those in the low or very low categories are considered food insecure.

Height and weight were self-reported. Physical activity level was self-assessed with two questions: 1) Do you consider yourself a physically active person? (yes/no) 2) Are you physically active (choose one) < 30 minutes/day or ≥ 30 minutes/day? Immediately upon completion, *About Being Active* was evaluated for interest, usefulness, timing, and format using a survey that included opportunity for open-ended comments, which was tested for face and content validity with the target audience [23]. In addition, reasons for participation in the randomized controlled study were examined for relationship to *About Being Active* participation. The Office for Research Protections of the Pennsylvania State University reviewed and approved this study. Consent was obtained by an online selection to participate.

### Data analysis

Mean ecSI/LI scores were compared among groups (e.g. BMI categories, perceived as physically active) using t-tests or analysis of variance as appropriate. EC (as designated by an ecSI/LI score ≥ 32) [7] or ecSI/LI score divided into tertiles were analyzed with Chi Square to examine association with other categorical variables (e.g., nutrition assistance program use, race). Influence of age, BMI, or weight satisfaction on ecSI/LI score differences according to physical activity categories was examined using Type III sums of squares in a univariate general linear model with ecSI/LI score as the dependent variable, either the perception of being physically active or of time of being physically active as fixed factors and each continuous variable of interest as a covariate. Categorical variables of interest, e.g. BMI categories or education level were included in the univariate general linear model as fixed factors to assess for interaction. Estimated marginal (unweighted) means and standard errors were reported for all general linear model analyses. *About Being Active* could be completed in one of 5 orders (first to last); in addition to examining for influence by specific order, categories were collapsed to enable comparison of those who completed it as the first or second lesson with participants who completed it as their third, fourth, or last lesson. System recorded time spent on the lesson was analyzed as a continuous variable as well as divided into three categories (< 5 minutes, 5 – 14.9 minutes, ≥ 15 minutes) based on congruence with projected time allotment (which was 15 minutes) and actual performance. Analyses were completed with SPSS 19.0.0, 2010 (IBM, Armonk, NY). P values < 0.05 were considered statistically significant.

Verbatim transcripts of participant comments were independently reviewed by two researchers to identify themes and to compare responses according to food security categories, SNAP use, EC status, or attributes of lesson completion (e.g., order or time). Conclusions derived from independent examination were iteratively compared and discussed.

## Results

### Participant characteristics

Participants were predominantly white (n=461, 92%; black n=19, 4%) and Non-Hispanic (n=489, 96%) and represented all geographic regions of Pennsylvania. More than one race choice was selected by 3% (n=15) of the sample. Average age was 30.7 ± 7.5 years for the total sample (n=512) and 31.6 ± 7.9 for those completing *About Being Active*. Being physically active < 30 minutes/day was associated with a lower education level. More than half had some college education and reported they were married or living with a partner. Supplemental Nutrition Assistance Program participation in the past year was reported by most participants; 64% of the sample was recruited from the SNAP participant list. More than 60% were either overweight or obese. A majority accessed the internet from home at least once each day. Participants reported internet access at work, a friend or neighbor’s house, family member’s home, library, or other. Participant characteristics including internet usage are shown in Table 2.

**Table 2:** Participant characteristics

**Characteristic **^**1**^	**Total sample (n=512) **^**2 **^**n (%)**	**Physically active **^**3 **^**n= 390 (%)**	**Not physically active **^**3 **^**n=122 (%)**	**Physically active ≥ 30 min/day **^**4 **^**n=362 (%)**	**Physically active < 30 min/day **^**4 **^**n=150 (%)**
**Identified with race/ethnicity choice (May Select > 1)**					
Hispanic/Latino	19 (4)	15 (4)	4 (3)	14 (4)	5 (3)
American Indian/Alaskan Native	8 (2)	4 (1)	4 (3)	2 (1)	6 (4)
Asian	3 (1)	3 (1)	0 (0)	3 (1)	0 (0)
Black/African American	30 (6)	22 (6)	8 (7)	16 (4)	14 (9)
Native Hawaiian/Pacific Islander	2 (< 1)	1 (< 1)	1 (1)	2 (1)	0 (0)
White	475 (93)	364 (93.3)	111 (91)	338 (93)	137 (91)
**Education level **^ns, **^					
< High school	36 (7)	30 (87)	6 (45)	33 (9)	3 (2)
High school grad/GED	180 (35)	139 (36)	41 (37)	130 (36)	50 (33)
Some college/2 yr degree	168 (33)	130 (33)	38 (31)	120 (33)	48 (32)
4 yr. College degree	126 (25)	89 (23)	37 (30)	77 (21)	49 (33)
**Marital status**					
Married/Living with partner	265 (52)	197 (51)	68 (56)	181 (50)	84 (56)
Separated/Divorced	81 (16)	62 (16)	19 (16)	56 (16)	25 (17)
Widowed	8 (2)	7 (2)	1 (1)	7 (2)	1 (1)
Never married	156 (31)	122 (31)	34 (28)	116 (33)	40 (26)
**SNAP Participation **^5^	273 (60)	209 (60)	64 (59)	199 (61)	74 (56)
**Parent**	403 (79%	301 (77)	102 (84)	283 (78)	120 (80)
Eating competence ^***, **^					
EC (ecSI ≥32)	190 (39)	164 (44)	26 (22)	151 (43)	39 (28)
Not EC (ecSI <32)	302 (61)	212 (56)	90 (78)	200 (57)	102 (72)
EC Tertile ^***, ***^					
Low tertile	144 (29)	89 (24)	55 (47)	83 (24)	61 (43)
Middle tertile	172 (35)	135 (36)	37 (32)	128 (36)	44 (31)
High tertile	176 (36)	152 (40)	24 (21)	140 (40)	36 (26)
BMI Category ^***, ***^					
Underweight (<18.5)	12 (2)	11 (3)	1 (1)	11 (3)	1 (1)
Normal weight (18.5 -24.9)	192 (38)	172 (44)	20 (16)	161 (44)	31 (21)
Overweight (25.0–29.9)	131 (26%	99 (25)	32 (26)	91 (25)	40 (27)
Obese ≥ 30	177 (35)	108 (28)	69 (57)	99 (27)	78 (52)
How satisfied are you with your current weight? ^***, ***^					
Very satisfied	32 (6)	28 (7)	4 (3)	29 (8)	3 (2)
Satisfied	74 (15)	68 (17)	6 (5)	64 (18)	10 (7)
Neutral	101 (20)	92 (24)	9 (7)	85 (24)	16 (11)
Unsatisfied	151 (30)	120 (31)	31 (25)	107(30)	44 (29)
Very unsatisfied	154 (30)	82 (21)	72 (59)	77 (21)	77 (51)
Food security status ^*, ns^					
High food security	194 (42)	160 (46)	34 (31)	145 (44)	49 (37)
Marginal food security	87 (19)	63 (18)	24 (22)	62 (19)	25 (19)
Low food security	77 (17)	52 (15)	25 (23)	54 (17)	23 (17)
Very low food security	101 (22)	75 (21)	26 (24)	66 (20)	35 (27)
**Do you ever worry about not having enough money to buy food? **^ns, **^					
Always	58 (11)	36 (10)	17 (14)	36 (10)	22 (15)
Often	75 (15)	49 (14)	24 (20)	49 (14)	26 (17)
Sometimes	152 (30)	108 (30)	33 (27)	108 (30)	44 (29)
Rarely	122 (24)	101 (28)	20 (16)	101 (28)	21 (14)
Never	104 (20)	67 (19)	28 (23)	67 (19)	37 (25)
**Usual internet access**					
Home	393 (77)	304 (78)	89 (73)	280 (77)	113 (75)
Work	40 (8)	26 (7)	14 (12)	23 (6)	17 (11)
Friend/Neighbor	11 (2)	8 (2)	3 (3)	7 (2)	4 (3)
Family member’s home	17 (3)	13 (3)	4 (3)	13 (4)	4 (3)
Library community center	38 (7)	29 (7)	9 (7)	30 (8)	8 (5)
Other	13 (3)	10 (3)	3 (3)	9 (3)	4 (3)
**Internet use frequency**					
At least once a day	392 (77)	298 (76)	94 (77)	271 (75)	121 (81)
A few times/week	102 (20)	77 (20)	25 (21)	75 (21)	27 (18)
A few times/month	14 (3)	11 (3)	3 (3)	12 (3)	2 (1)
A few times/year	4 (1)	4 (1)	0 (0)	4 (1)	0 (0)

* P < 0.05, ** P ≤ 0.01, *** P ≤ 0.001, ^ns^ P > 0.05.

^1^ Statistical significance marks first compare being physically active or not, then compare being physical activity ≥ 30 min/day or < 30 min/day.

^2^ Sample size may vary due to missing responses.

^3^ Based on participant response to the question, “Do you consider yourself a physically active person? Yes or No?”.

^4^ Based on participant response to the question, 'Are you physically active 30 minutes or more per day or < 30 minutes per day?”.

^5^ Supplemental Nutrition Assistance Program.

Response fidelity was evident. For example, amount of desired weight loss was significantly inversely correlated to weight satisfaction (r = -.58, n=458, P < 0.001) and worry about having enough money for food correlated with food insecurity as measured by the USDA Food Security Screener (r= .55, n=501, P < 0.001).

### Relationship between physical activity and demographic and psychosocial characteristics

Perceived physical activity levels were assessed by asking participants if they believed they were physically active, and also by inquiring into whether or not they were physically active for more than 30 minutes per day. A majority (76%) perceived themselves to be physically active and 71% reported being physically active ≥ 30 minutes per day. However, weight dissatisfaction was reported by 60%; weight satisfaction was significantly (P< 0.001, r =0.6) correlated to a lower BMI.

**Figure 2:**
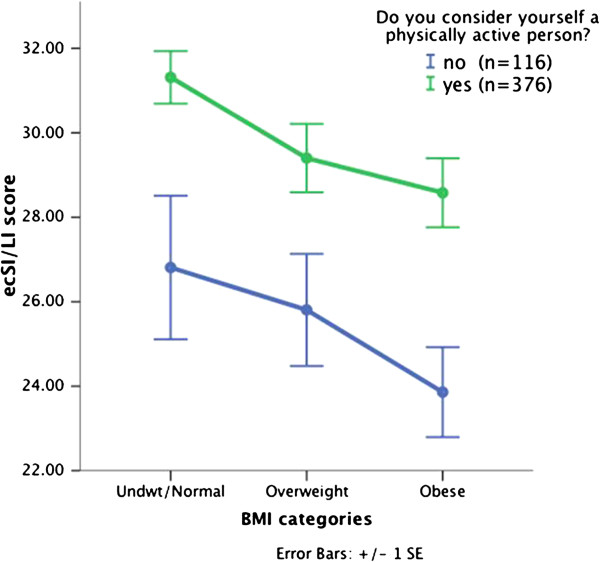
**ecSI/LI scores compared by physical activity status and BMI categories.** Difference between those who perceive being physically active and those who don’t P<0.001; Difference among 3 BMI categories P=0.042.

Those who considered themselves to be physically active were more likely to be younger, of normal weight and to be satisfied with their current weight (Table 3). In addition, wanting to lose 25 pounds or more was significantly associated with not being physically active (Chi Square 33.9, P<0.001). Although 43% (n= 164) of physically active participants wanted to lose 25 pounds or more, a wish for this level of weight loss was reported by 74% (n=89) of inactive participants. Those reporting being physically active also were more likely to be eating competent (43.5% vs. 22.4%, Chi Square 16.8, P<0.001) and to be in the high EC tertile (40.4% vs. 20.7%, Chi Square 27.1, P<0.001). Compared to those who reported not being physically active, ecSI/LI scores were significantly higher in those who perceived being physically active (Table 3). Also, all attributes of EC were higher with all ecSI/LI subscale scores significantly greater in those reporting being physically active (all P ≤ 0.002; data not shown). This relationship persisted when controlling for BMI (29.9 ± .4 vs. 25.5 ± .8; F=23.1, P<0.001), weight satisfaction (29.6 ± .8 vs. 25.4 ± .4; F=13.3, P < 0.001), and age (30.0 ± .4 vs. 25.1 ± .7;F=31.5, P <0.001). Furthermore, although ecSI/LI scores increased from obese to overweight to normal BMI categories regardless of physical activity status and were significantly different between obese and normal BMIs (P=0.013), the lowest score for those perceiving being physically active (i.e. BMI categorized as obese) was higher than that for normal weight participants who weren’t physically active (Figure 2).

**Table 3:** **Comparisons based on reported physical activity levels (n=512) **^**ab**^

**Variable**	**Total sample (n=512) (Mean ± SD)**	**Physically active **^**1 **^**(n= 390)**	**Not physically active (n=122)**	**Physically active ≥ 30 min/day **^**2 **^**(n=362)**	**Physically active < 30 min/day (n=150)**
**BMI**	28.3 ± 7.2	27.1 ± 6.5	32.4 ± 7.9***	27.1 ± 6.5	31.4 ± 7.9***
**Weight satisfaction **^**3**^	3.6 ± 1.2	3.4 ± 1.2	4.3 ± 1.0***	3.4 ± 1.2	4.2 ± 1.0***
**EC Score **^**4**^	28.9 ± 8.5	30.1 ± 8.3	24.9 ± 8.1***	29.9 ± 8.3	26.3 ± 8.6***
**Age (years)**	30.7 ± 7.5	30.3 ± 7.4	32.0 ± 7.8*	30.2 ± 7.3	32.0 ± 7.8**

*P≤0.05, **P≤0.01, ***P≤ 0.001.

^1^ Based on participant response to the question, “Do you consider yourself a physically active person? Yes or No?”.

^2^ Based on participant response to the question, “Are you physically active 30 minutes or more per day or < 30 minutes per day?”.

^3^ Possible score 1(Very satisfied) to 5 (Very unsatisfied).

^4^ Scores range from 0 to 48; higher scores indicate greater eating competence; total n=492.

^a^ Sample size may vary due to missing responses.

^b^ Statistical tests are between physically active and not physically active or between physically active 30 minutes or more per day and < 30 minutes per day.

### Relationship between perceived levels of physical activity and demographic and psychosocial characteristics

**Figure 3:**
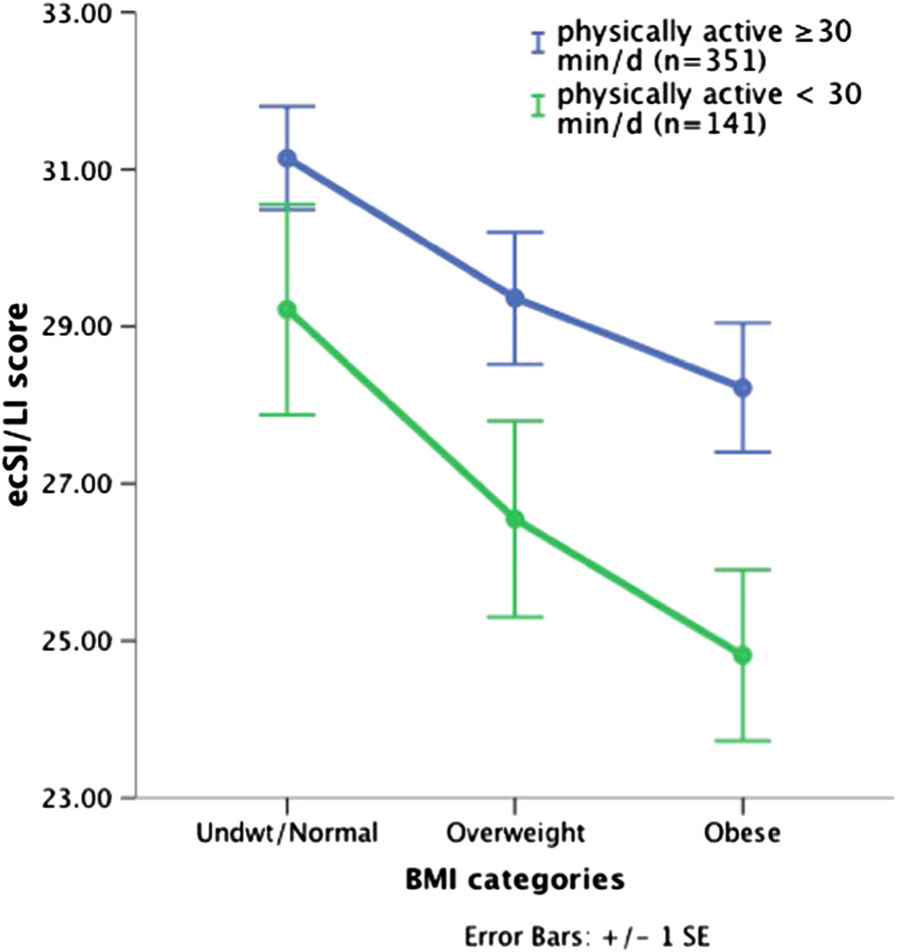
**ecSI/LI scores compared by amount of physical activity and BMI categories.** Difference between those who perceive being physically active < 30 minutes/day and those are physically active 30 minutes or more each day P<0.001; Difference among 3 BMI categories P<0.001.

Participants who reported being physically active 30 minutes or more per day gave a higher rating to their eating habits (P<0.001), worried less about having enough food (P=0.008), were more educated (P=0.04) and were younger, with a lower BMI, and greater weight satisfaction. Perception of being physically active 30 or more minutes a day rather than < 30 minutes was associated with being eating competent (43% vs. 27.7%, Chi Square 10.1, P=0.002) and in the high EC tertile (39.3% vs. 25.5%, Chi Square 19.8, P<0.001). Compared to those who reported being physically active < 30 minutes a day, ecSI/LI scores were significantly higher in those who perceived being physically active ≥ 30 minutes a day (Table 3). All ecSI/LI subscale scores were significantly greater in those reporting being physically active ≥ 30 minutes a day (all P ≤ 0.003; data not shown). This relationship remained significant when controlling for BMI (29.7 ± .4 vs. 26.8 ± .71; F=11.52, P=0.001), weight satisfaction (29.3 ± .4 vs. 27.6 ± .7, F=4.43, P=0.04), age (29.8 ± .4 vs. 26.5 ± .7, F=15.7, P<0.001) and having a post-secondary education (29.9 ± .5 vs. 26.3 ± .8; F=17.2, P<0.001). As shown in Figure 3, the pattern of ecSI/LI scores decreasing from normal to obese BMI categories for both physically and not physically active participants was also noted when comparing those who reporting being physically active ≥ 30 minutes and < 30 minutes daily.

### Response to *About Being Active*

Of 204 who started *About Eating*, 168 (82%) completed the *About Being Active* lesson, with time stamp and order of lesson completion recorded for 164 participants. Of those completing *About Being Active*, 145 could choose to complete it in any order (i.e., 19 were in the group with requisite completion of this lesson last), and of these, 23 (16%) completed it first, 19 (13%) second, 13 (9%) third, 13 (9%) fourth and 77 (53%) completed it last. Order of lesson completion was not associated with responses to lesson features, self-reported physical activity status, BMI, EC, weight satisfaction, emotional or uncontrolled eating behaviors, or desired weight loss. Participants completing *About Being Active* first or second (rather than later) tended to spend more time on the lesson (10.3 ± 7.1 vs. 7.6 ± 8.1 min; t=1.9, P=.056) without a greater amount of time spent on the *About Eating* program. Of the 42 who completed *About Being Active* first or second, 21% (n=9) spent 15 minutes or more viewing it compared to only 9% of the 122 (n=11) who completed it as a 3^rd^, 4^th^, or 5^th^ lesson (Chi Square 9.71, P=0.008). Although not statistically significant, of note is that participating in *About Eating* because of an interest in weight loss was denoted by 33% of those selecting *About Being Active* as a first or second lesson; only 25% of those completing the lesson later identified weight loss as a rationale for participation. However, wanting to lose 25 or more pounds was not associated with spending more time on *About Being Active* or seeking it out as the first or second lesson.

According to the computerized time stamp, participants spent an average of 8.2 ± 7.6 minutes at the lesson website (range 1 – 53 minutes) and most thought overall lesson length was good. Time spent on the lesson was not significantly different between those identifying themselves as physically active or not (8.0 ± 6.9 vs. 8.8 ± 9.6) or as being active ≥ 30 min/day compared to < 30 min/day(8.3 ± 7.2 min vs. 7.8 ± 8.3 min/day). Responses to lesson features were not related to time spent on *About Being Active*, EC status or ecSI/LI score tertile.

Overall, lesson feedback was positive; the pictures and overall design and/or color were liked by most participants (Table 4). A majority disagreed with the statement that the lesson was difficult to read and that navigating the website was difficult. Only 1.4% of the derogatory evaluation choices were selected (n=17 selections of 1176 possible, i.e. 168 participants X 7 questions). These 17 responses were from 12 participants. (1 participant provided 5 negative responses, which were not for readability or website navigation, and another participant was negative about lesson usefulness and interest). Both participants denoted being physically active, for 30 or more minutes each day, neutral about their weight satisfaction, and were older than 37 years. The 12 participants with negative lesson responses were compared with the total sample for perceived physical activity, amount of physical activity, EC status, and factors associated with physical activity and EC, i.e., BMI category, weight satisfaction, and age. Negative responders were similar to the total sample in age (mean 33.8 years), weight satisfaction (50% were very/unsatisfied, 25% very/satisfied), perceiving being physically active (83%) for 30 or more minutes each day (75%) and EC status (42% eating competent, 42% high EC tertile).

**Table 4:** Response to lesson content and design

**Response**	**Total sample (n=168) **^**1 **^**n (%)**	**Physically active **^**2 **^**n= 131 (%)**	**Not physically active **^**2 **^**n=37 (%)**	**Physically active ≥ 30 min/day **^**3 **^**n=115 (%)**	**Physically active < 30 min/day **^**3 **^**n=53 (%)**
**The lesson was difficult to read**					
Sometimes	1 (1)	1 (1)	0 (0)	1 (1)	0 (0)
No, not very much	25 (15)	17 (13)	8 (22)	13 (1)	12 (23)
No, not at all	140 (84)	112 (86)	28 (78)	100 (88)	40 (77)
**Getting around the website was difficult**					
Yes, definitely	1 (1)	1 (1)	0 (0)	1 (1)	0 (0)
Yes	2 (1)	1 (1)	1 (3)	1 (1)	1 (2)
Sometimes	2 (1)	1 (1)	1 (3)	1 (1)	1 (2)
No, not very much	21 (13)	16 (13)	5 (15)	12 (11)	9 (18)
No, not at all	136 (84)	109 (85)	27 (79)	98 (87)	38 (78)
**This lesson was interesting**					
Yes, definitely	81 (49)	64 (49)	17 (47)	51 (45)	30 (58)
Yes	74 (45)	58 (45)	16 (44)	55 (48)	19 (37)
Sometimes	7 (4)	4 (3)	3 (8)	4 (4)	3 (6)
No, not very much	1 (1)	1 (1)	0 (0)	1 (1)	0 (0)
No, not at all	3 (2)	3 (2)	0 (0)	3 (3)	0 (0)
**This lesson was useful to me**					
Yes, definitely	84 (50)	67 (51)	17 (47)	52 (45)	32 (62)
Yes	74 (44)	56 (43)	18 (49)	55 (48)	19 (37)
Sometimes	6 (4)	5 (4)	1 (3)	5 (4)	1 (2)
No, not very much	1 (1)	1 (1)	0 (0)	1 (1)	0 (0)
No, not at all	2 (1)	2 (2)	0 (0)	2 (2)	0 (0)
**I liked the pictures**					
Yes, definitely	57 (34)	47 (36)	10 (28)	34 (30)	23 (44)
Yes	82 (49)	62 (47)	20 (56)	64 (56)	18 (34)
Sometimes	24 (14)	19 (15)	5 (14)	15 (13)	9 (17)
No, not very much	3 (2)	2 (2)	1 (3)	1 (1)	2 (4)
No, not at all	1 (1)	1 (1)	0 (0)	1 (1)	0 (0)
**Overall, the length of the lesson was good**					
Yes, definitely	79 (47)	62 (47)	17 (47)	47 (41)	32 (62)
Yes	86 (51)	67 (51)	19 (53)	66 (57)	20 (38)
Sometimes	1 (1)	1 (1)	0 (0)	1 (1)	0 (0)
No, not at all	1 (1)	1 (1)	0 (0)	1 (1)	0 (0)
**I liked the overall design and/or color**					
Yes, definitely	66 (40)	53 (41)	13 (36)	41 (36) ^a^	25 (49)
Yes	87 (52)	69 (53)	18 (50)	68 (59)	19 (37)
Sometimes	12 (7)	7 (5)	5 (14)	5 (4)	7 (14)
No, not at all	1 (1)	1 (1)	0 (0)	1 (1)	0 (0)

^1^ Sample size may vary due to missing response.

^2^ Based on participant response to the question, “Do you consider yourself a physically active person? Yes or No?”

^3^ Based on participant response to the question, "Are you physically active 30 minutes or more per day or < 30 minutes per day?”.

^a^ Chi Sq.=9.6, P=.023, Represents comparisons between physically active ≥ 30 min/day vs. physically active < 30 min/day.

Participants responding to the open-ended invitation to comment on the lesson (n=48) offered mostly positive responses. For example, one remarked that she learned the most from this lesson. Another stated, “I thought the lesson was very useful in clearing up the "myths" on what I believed was true. I am planning on using this to help me become more active and also help my children become more active.” Participants expressed interest in applying what they had learned and indicated they were motivated to increase their time spent in physical activity. Use of physical activity as a strategy to reduce stress and fatigue was reported. One participant commented that the most important point she learned was, “being more active will fight fatigue” and another stated, “I didn’t realize that working out would lessen my stress.” Participants liked the interactive components of the lesson, including the quizzes and activities. As reported for the choices made from the evaluation scales, negative comments were rare. One participant stated, “I felt like the activity chart/graph that was printable was very complex…A simpler chart may have been less intimidating.” Researchers unanimously agreed that comments didn’t differ by SNAP status, food security status, EC, order of lesson completion or amount of time spent on the lesson.

### Response to *About Being Active* components: case studies and intra-lesson quiz

A lesson component included four case studies to demonstrate different daily activity patterns. Participants were asked to select with whom they could most relate on a typical day (i.e., sit-down Sarah, lifestyle Linda, hardcore Hayley, and combo Chris). More (45%) reported being most like “Lifestyle Linda,” a person who was physically active throughout the day by participating in “lifestyle” activities, such as household chores. Participants were surprised that they didn’t have to exercise all at once, and they reported this new information encouraged them to increase their current level of physical activity. They were interested that household chores contributed to their daily physical activity. Only 7% of participants indicated that they related to “Hardcore Hayley”, a person who exercised vigorously at the gym one or two times during the day. This exercise pattern wasn’t realistic for participants with money, time, and transportation constraints that make gym access difficult. Of interest is that a significantly (both P<0.001) greater number of participants who reported not being active or being active < 30 minutes a day identified with sit-down Sarah (67% vs. 22% for those who perceive being physically active; 58% vs. 20% for those denoting being active 30 or more minutes per day).

*About Being Active* included a 6-item quiz on knowledge of physical activity concepts, such as the use of sports drinks during exercise and exercise intensity. Mean score (out of 6 possible) was 3.9 ± 1.1 (median 4.0; range 0 – 6). Quiz scores were not significantly different between those who considered themselves to be physically active and those who did not or between those who reported being active 30 or more minutes a day and those less active. Therefore, the perception of being physically active was not associated with greater knowledge. In addition, quiz scores were not significantly different between those who completed *About Being Active* early (first or second lesson) or later in the program and were not associated with amount of time spent on the lesson, EC status or ecSI/LI score tertile.

## Discussion

SNAP participation, food insecurity, and worry about money for food were all high in this sample, indicating the women were low-income. As anticipated, 60% or more did not have a 4 year college degree, were either overweight or obese and expressed dissatisfaction with their weight. Internet access was an inclusion criterion, however usage frequency was high (i.e., 77% accessed the internet several times a day), and paralleled other studies with low-income persons [19,20]. ecSI/LI scores and proportion identified as eating competent were strikingly similar to other studies with low-income participants [3,7,26].

EC was clearly associated with self-reported physical activity in this sample of low-income women. Women who were not physically active were less likely to be eating competent and were in the lowest tertile of ecSI/LI scores. The fact that physically active women were more likely to be eating competent suggests that EC reflects a global model, not limited to eating behavior. EC implies capability, autonomy, self-control and self-awareness. These traits, which suggest intrapersonal support, have distinguished physically active from inactive persons in economically disadvantaged samples [27,28]. In addition, enjoyment, confidence, intrinsic motivation and autonomous regulation, all components of ecSatter, are consistently correlated with regular participation in physical activity [15]. Findings from studies of EC and physical activity in university students support this concept. The eating attitudes subscale, which denotes a vigorous and vital approach to eating, is significantly (P<0.001) associated with a higher VO2max. Contextual skills subscale scores are similarly related (P<0.001) to amount of moderate and vigorous physical activity as denoted on the IPAQ; both scales measure behaviors that require planning, time management, and goal directed behaviors. Thus, interventions that increase EC may indirectly enhance readiness to be more physically active.

This online physical activity lesson was well received by low-income women. We anticipated being able to identify and distinguish proponents of *About Being Active* to assist with revision and further development. However, BMI, prior knowledge, enthusiasm to complete the lesson and time spent on the lesson were not related to overall impression, possibly as a result of the nearly uniform applause for *About Being Active*. Although this lesson was part of a larger curriculum of five lessons, participant comments suggest that *About Being Active* could be offered as a stand-alone lesson or incorporated with other physical activity programs. Participants reported learning a lot from the messages presented in the short lesson, indicating that this lesson would be conducive to the time constraints experienced by this population. Participants showed interest in the lesson content and expressed excitement in applying what they had learned into their everyday lives. *About Being Active* also has potential to provide impact beyond the individual because participants also planned to apply what they had learned to their family lifestyle.

A limitation of the study is that the level of physical activity in this study was based on self-report and self-perception, thereby tempering conclusions that relate physical activity and EC. However, numerous studies about physical activity in low-income samples utilized self-reported responses to provide physical activity profiles and suggestions to address barriers, motivators, and educational needs. The proportion reporting being physically active in these studies closely approximates the level of 76% reported here [13,15,16,20,27,28]. The WISEWOMAN intervention assessment utilized only two questions, both requiring either a yes or no response [16]. In addition to self-report being a standard research practice, soundness of self-classifying physical activity status was supported by the significant relationship between identifying with “Sit-down Sarah” and answering “No” to being physically active or being active < 30 minutes. However, further research on how to conveniently and accurately assess physical activity status would be helpful. Some discrepancy in the definition of physical activity has been reported and a standardized approach to the inclusion of housework and child care as physical activity would benefit research. A limitation to the *About Being Active* evaluation is the lack of follow-up to determine if the intentions to increase physical activity noted in the comments actually translated into behavior. Educational program development will benefit from further research to better understand the dynamics between EC and physical activity and how perception of being physically activity (or not) relates to motivation to seek related education.

## Conclusion

Overall eating competence was low in this sample of low-income women and was higher for normal weight than overweight and obese women. However, women with perception of being physically active or physically active for 30 minutes or more each day were more EC. Obese physically active women were more EC than normal weight women who did not report being physically active. Low income women were interested in learning about physical activity and responded positively to online delivery of a lesson designed to enhance physical activity. Additional research is needed to determine if EC is associated with responses to physical activity education.

## Competing interests

The authors declare that they have no competing interests.

## Authors’ contributions

BL designed the study, developed the survey sets, supervised recruiting, conducted data analyses and drafted the manuscript. PW designed and supervised the interface for the online study components and supervised database development and management. KA coordinated recruitment and participant contact, prepared tables and figures, and assisted with manuscript preparation. All authors read and approved the final manuscript.

## Pre-publication history

The pre-publication history for this paper can be accessed here:

http://www.biomedcentral.com/1472-6874/13/12/prepub
